# Advancing food safety risk assessment in China: development of new approach methodologies (NAMs)

**DOI:** 10.3389/ftox.2023.1292373

**Published:** 2023-11-17

**Authors:** Daoyuan Yang, Hui Yang, Miaoying Shi, Xudong Jia, Haixia Sui, Zhaoping Liu, Yongning Wu

**Affiliations:** NHC Key Laboratory of Food Safety Risk Assessment, China National Center for Food Safety Risk Assessment, Beijing, China

**Keywords:** next-generation risk assessment, new approach methodologies, China, China National Center for Food Safety Risk Assessment, food toxicology

## Abstract

Novel techniques and methodologies are being developed to advance food safety risk assessment into the next-generation. Considering the shortcomings of traditional animal testing, new approach methodologies (NAMs) will be the main tools for the next-generation risk assessment (NGRA), using non-animal methodologies such as *in vitro* and *in silico* approaches. The United States Environmental Protection Agency and the European Food Safety Authority have established work plans to encourage the development and application of NAMs in NGRA. Currently, NAMs are more commonly used in research than in regulatory risk assessment. China is also developing NAMs for NGRA but without a comprehensive review of the current work. This review summarizes major NAM-related research articles from China and highlights the China National Center for Food Safety Risk Assessment (CFSA) as the primary institution leading the implementation of NAMs in NGRA in China. The projects of CFSA on NAMs such as the Food Toxicology Program and the strategies for implementing NAMs in NGRA are outlined. Key issues and recommendations, such as discipline development and team building, are also presented to promote NAMs development in China and worldwide.

## 1 Introduction

The food safety risk assessment is an important component in risk analysis and provides scientific support for activities of risk management. As reviewed by Wu et al. (2018), the national food safety risk assessment system of China has been developed since 2009. Established in 2011, China National Center for Food Safety Risk Assessment (CFSA) is the only technical institution for food safety risk assessment at the national level in China, under the direct leadership of the National Health Commission of the People’s Republic of China. CFSA serves as the secretariat of the National Expert Committee for Food Safety Risk Assessment, and one important responsibility of CFSA is to implement food safety risk assessment projects and provide technical support and policy-making suggestions to the related government departments.

Toxicology is the basis of food safety risk assessments. However, considering the large number and complex toxicological effects of food chemicals, traditional animal tests are costly, time-consuming, and labor-intensive to identify the hazards of food chemicals. Establishing alternative, highly sensitive, and high-throughput methods are the primary technical needs. In recent years, scientific and technological progress, especially the rapid development in the life sciences, has provided new opportunities for the development of toxicological research methods. In 2007, the United States National Research Council proposed that the toxicity in the 21st century would be transformed from *in vivo* experiments to alternative *in vitro* assays to assess health risks (Krewski et al., 2010). Using alternative technologies such as *in vitro* cell tests to achieve risk assessment is based on the in-depth understanding of the toxicological mechanisms of chemicals, including molecular and cellular levels of toxicity pathways, tissue and organ levels of toxicity effects, and then adverse outcome pathways at individual and population levels. This requires collecting scientific evidence at various levels (Escher et al., 2022). Based on this concept of toxicological testing development, food toxicology is also facing a transition from traditional animal experiments to new strategies based on *in vitro* and *in silico* testing.

On the other hand, people’s awareness of animal welfare protection has reached a historical height. As more countries and organizations are committed to the 3R principles (replacement, reduction, and refinement) of animal testing, the prohibition of animal experiments and their replacement by other experimental methods are the needs of the times and future trends. In this regard, Europe is at the forefront. As early as 1991, the European Center for the Validation of Alternative Methods (ECVAM) (renamed the EU Reference Laboratory for Alternatives to Animal Testing, EURL ECVAM in 2011) was established to reduce animal testing. In 2010, the EU directive 2010/63/EU on the protection of laboratory animals stated that member states should aim to develop and validate alternative methods (Directive, 2010). In 2012, the Cambridge Declaration stated that all non-human animals have a biological basis for consciousness (Low et al., 2012). In 2013, the EU took the lead in implementing a complete ban on cosmetics animal testing globally. Norway, New Zealand, Israel, India, and other countries are also following up (Sreedhar et al., 2020).

In 2020, the European Commission issued the Chemicals Strategy for Sustainability to promote interdisciplinary research and digital innovation focusing on advanced analytical tools, methods, models, and data analysis capabilities, thereby reducing animal experimentation (Commission, 2020). In 2021, European Food Safety Authority (EFSA) released Strategy 2027, advocating the promotion of new approach methodologies (NAMs) based on alternatives to animal experiments, the development of cutting-edge new methods, and the improvement of the quality and efficiency of hazard risk assessment of chemicals or food-related substances (EFSA, 2021). Compared with Europe, the strategic goals and initiatives of the United States to support animal alternative technologies are more explicit. In 2019, the United States Environmental Protection Agency (EPA) released phased goals to reduce animal testing and will stop conducting or funding research on mammals by 2035 (Grimm, 2019). In 2022, the United States Food and Drug Administration (FDA) announced that new drugs will no longer require animal testing before human clinical trials, but will rely more on computer modeling, organ chips, and other new methods developed in recent years (Wadman, 2023). The regulatory prohibition of animal experiments may soon extend to food and pharmaceuticals.

Given the above reasons, the non-animal based NAMs that include emerging technologies for the next-generation risk assessment (NGRA) are the focus worldwide. Most NAMs are toxicologically related, including *in vitro* alternative methods, extrapolation based on physiologically based toxicokinetic (PBTK), and computational toxicological methods. Other NAMs cover the rest aspects of NGRA such as exposome applied in the exposure assessment and integrated approaches for testing and assessment (IATA) method used to comprehensively integrate all the related information from different methods for a specific risk assessment scenario. The United States EPA and EFSA have established work plans to encourage the development and application of NAMs in NGRA (EPA, 2021; Escher et al., 2022). In 2023, EFSA also reviewed its activities taken to implement NAMs in food safety risk assessments (Cattaneo et al., 2023). In support of the European Union’s Chemicals Strategy for Sustainability and the European Green Deal’s “Zero pollution”, a Partnership for the Assessment of Risks from Chemicals (PARC) has been established involving approximately 200 institutions from 28 countries as well as the European Chemical Agency (ECHA), EFSA, and the European Environment Agency (EEA), for the development of next-generation chemical risk assessment to protect human health and the environment (Partnership for the Assessment). Most NAMs are still in the research stage rather than in regulatory risk assessment. China is also developing NAMs for NGRA, and CFSA is the primary institution leading the implementation of NAMs in NGRA in China. This review provides an overview of the work of CFSA related to NAMs and summarizes major NAM-related research articles from China. This review also describes the key issues and the corresponding countermeasures of CFSA to develop and apply NAMs in food safety risk assessment.

## 2 Progress in China

The food supply and consumption scale of China are huge, with complex structures covering all aspects from agricultural production to food processing. The potential health hazards are not only numerous in number but also often show unique characteristics of China’s food safety. Since the establishment of the national food safety risk assessment system, achievements have been made in the development of methodology and procedure, data collection, team building, and the accomplishment of a series of risk assessment projects (Wu et al., 2018).

As toxicology is the basis for hazard assessment in food safety risk assessment, CFSA officially proposed the “Food Toxicology Program” in 2013 and began to organize the development and application of new technologies based on the relevant framework. The “Food Toxicology Program” is a basic, systematic, and long-term work. This program not only strengthened the development of traditional food toxicology in China but also promoted the development of NAMs. Specifically, by implementing this program in China, 1) China’s food safety emergency response capabilities were increased, and scientific guarantees were provided for food safety risk assessment and risk management by establishing a systematic toxicological database and continuously accumulating toxicological information on chemicals; 2) hazard assessments of certain chemical substance were based on the toxicological data generated from this program, thereby enabling China’s food toxicology to have a say in the international community and play a leading role in the risk assessment and standard setting of substances unique to China; 3) research on new toxicological NAMs can be carried out to explore and establish various feasible key technologies for hazard assessment.

After 10 years of development, the “New Risk Identification and Toxicity Screening New Technology Platform” has gradually established from scratch, covering methods including toxicological threshold of concern (TTC), quantitative structure-activity relationship (QSAR), rapid detection technologies based on human cell and model organisms, alternative toxicological methods, as well as systematic toxicological methods represented by various omics (toxicogenomics, toxic transcriptomics, toxic metabolomics, etc.) and translational toxicological methods represented by adverse outcome pathways (AOPs).

The progress made including the NAMs development based on the “Food Toxicology Program” and some NAMs CFSA and research institutions in China are working on are listed below.

### 2.1 *In vitro* alternative methods

Based on the National Key R&D Program Project “Integrated Technology and Application Research on Hazard Assessment of Food Pollutants” (2018–2021), aiming at the key technical bottlenecks in the risk assessment of food pollutants in China, the CFSA toxicology team developed 10 hazard identification technologies, including human macrophage high-content models, human hepatocyte lipid toxicity models, human white-brown fat cell differentiation interference models, human embryonic stem cell developmental toxicity models, zebrafish liver toxicity models, and extended first-generation reproductive toxicity models, etc. Breakthrough innovations have been made in the research and application of alternative testing methods. In particular, comparable and verifiable rapid and efficient alternative testing toxicity and hazard identification techniques have been established for three toxicity endpoints with high international concern (carcinogenicity, reproductive developmental toxicity, and genotoxicity). This made up for the lack of alternative testing methods in China and promoted the shift in toxicity testing from traditional animal testing to alternative testing technologies.

To achieve rapid and accurate screening of endocrine disrupting toxicity in food related chemicals, the CFSA research team has established a high-throughput hazard identification *in vitro* alternative technology based on a model that employs differentiated macrophages transfected with PPRE receptor response elements to enhance the sensitivity of cell carrier signals. Through the series of upstream and downstream key molecules, the high-content analysis of multiple effect targets is realized. This model has been used for toxicity screening and classification of over 3,000 food compounds and 160 priority chemicals were identified, providing a scientific basis for subsequent hazard assessment or health effect studies. In addition, this technology found that the plasticizer diethylhexyl phthalate (DEHP) can activate macrophage peroxisome proliferator-activated receptors, interfere with lipid metabolism balance, and induce fatty liver (Xu et al., 2022a). Related research results were published in EHP and reported in the form of “Science Selection.” Peer review pointed out that “this study is an important progress in this field. We may have underestimated the role of macrophages in DEHP-induced fatty liver. This innovative discovery reflects the key role of the immune system in metabolic diseases” (Schmidt, 2022). On this basis, combined toxicity assessments of various phthalate plasticizers (DEHP, DBP, DIBP, DINP, DCHP, DMP, DEP) are currently under investigation.

For embryonic developmental toxicity, the CFSA laboratory has established a high-content hazard identification *in vitro* alternative model based on human embryonic stem cell embryoid bodies and differentiation of inner, middle, and outer germ layers (Fang et al., 2019). The toxicity of zearalenone and trichothecene were detected in the model and both showed toxic effects on the development of specific embryonic layers mechanisms (Fang et al., 2018; Cao et al., 2019). The developed model can be considered a promising tool to assess developmental toxicity and interpret the underlying mechanism.

Other cell-based and organoids *in vitro* methods were also developed in China for specific research purposes of food hazards. The *in vitro* Rijksinstituut voor volksgezondheiden milieu digestion model and Caco-2 cell model were used to assess the bioavailability and bioaccessibility of Cadmium in rice in China, suggesting the necessity to consider the bioavailability and bioaccessibility to accurately assess the health risk of cadmium exposure through rice (Yao et al., 2021; Deng et al., 2022). Retinal organoid models differentiated from human embryonic stem cells were used to explore the effects of bisphenols on the early development of the retina (Li et al., 2022a). Liver organoids and intestinal organoids differentiated from human pluripotent stem cells (hiPSCs) were used to analyze the hepatotoxicity of polystyrene (PS) microplastics and the accumulation of PS nanoplastics in intestinal organoids, respectively (Cheng et al., 2022; Hou et al., 2022). A murine small intestine organoid model was used to explore the effects of sunset yellow on small intestinal cells (Kong et al., 2021). Organoids from murine thyrocytes with thyroid follicle-like structures were developed to study the adverse effects of iodide excess on thyroids (Yu et al., 2023).

### 2.2 Extrapolation based on PBTK modeling

Given the ongoing research on NAMs, a wide range of *in vitro* biological assays has been developed to screen a chemical of interest for different toxicity endpoints or to elucidate the modes of action underlying the toxicity (Bernauer et al., 2005). Clearly, many *in vitro* experiments do not (yet) capture the toxicokinetics information, and thereby do not fully reflect the *in vivo* situation. To enable the use of *in vitro* assays in human risk assessment, a translation is required to convert the *in vitro* concentration-response data to *in vivo* human dose-response data, taking into account toxicokinetics (Blaauboer, 2010; Bell et al., 2018). As an important element of NAMs, PBTK models can link the internal concentrations upon exposure to known external doses of the chemicals (i.e., forward dosimetry), and they can also be used to extrapolate *in vitro* toxicity data to external dose values considering toxicokinetics, which is referred to as reverse dosimetry (Rietjens et al., 2011). CFSA is dedicated to developing a PBTK modeling-based hazard assessment strategy and actively promotes its application in food safety risk assessment. CFSA has implemented the PBTK models to define the human equivalent dose factor of Bisphenol A, as well as predicted human equivalent doses for several phenolic endocrine disrupting chemicals by using the PBTK modeling-based *in vitro* to *in vivo* extrapolation (IVIVE) approach (Xie et al., 2023). In addition, the application of PBTK modeling is further expanded to a more comprehensive compound-specific risk assessment, which provides more accurate hazard assessment information for the higher tier risk-based decision making. This is demonstrated by a case study where the PBTK modeling-based IVIVE approach was integrated with Monte Carlo simulation to quantify the inter-individual kinetic variation underlaying possible variation in cardiotoxicity of methadone, and to derive chemical-specific adjustment factors (CSAFs) for inter-individual and inter-ethnic kinetic differences for the Caucasian and Chinese populations (Shi et al., 2022). Though PBTK modeling has been used in a few regulatory risk assessment projects in China, CFSA is continuously working on the scientific and regulatory gaps, such as developing the guidance on when and how to apply PBTK modeling to replace part of the default uncertainty factors, how to better address the sensitivity of the input parameters, and developing the standardized methods for PBTK modeling, also pointed out in the roadmap for NAMs by EFSA (Escher et al., 2022).

Related studies by other research institutions in China included the case studies of using PBTK to predict liver toxicity induced by troglitazone (Yu et al., 2020) and acetaminophen (Zhang et al., 2020a), to predict cardiovascular toxicity induced by doxorubicin (Li et al., 2021a).

### 2.3 Computational toxicological methods

Computational toxicological methods such as QSAR and read across are also important *in silico* NAMs for the NGRA of food safety. CFSA has established the application guide of QSAR in food safety risk assessment and the application guide of read across in food safety risk assessment, including the overall principle, definition, application flow chart, and detailed explanation of the application procedure (CFSA, 2022). These guidelines serve as the basis for the application of these methods in the food safety risk assessment projects in China and the development of the next-generation computational toxicological methods.

However, QSAR and read across are currently more used in research in China, and they have not been formally applied to the food safety risk assessment projects by CFSA. Nevertheless, relevant research articles have been published by CFSA and various research institutions in China. Ma et al. (2021) used three QSAR tools (Toxtree, VEGA, T.E.S.T) and developed a risk classification strategy for chemicals migrated from food contact materials. Sun et al. (2021) developed QSAR models to predict the acute oral toxicity of polycyclic aromatic hydrocarbons (PAHs), which had been the focus of human health risk assessment due to their existence in foods and the potential toxicological effects. To study the toxicity mechanisms of halogenated disinfection byproduct (DBPs), Zhang et al. (2020b) developed a QSAR model using the *in vitro* cytotoxicity data of 15 halogenated aromatic DBPs tested with mammalian Chinese Hamster Ovary cells, and they found the interaction of aromatic DBPs with catalase and the electrophilic/nucleophilic reactivity were associated with the cytotoxicity. Found in foods and as a potential human health hazard, Hao et al. (2019) constructed QSAR models to predict the mutagenicity of nitroaromatic compounds. Wang et al. (2021) developed QSAR models to predict the peroxisome proliferator-activated receptor γ binding affinity of chemicals, to evaluate the potential endocrine-disrupting effects of chemicals. Xu et al. (2023) used the QSAR model to predict the bioconcentration factors of water pollutants without toxicity data.

Food contaminants such as PAHs and DBPs mentioned above are also either parts of the current priority risk assessment projects or have been considered as the potential projects of CFSA. But these contaminants contain a series of chemicals and are more difficult to assess their health risk compared to the single chemical contaminant, and one important reason is the lack of toxicological data. Therefore, QSAR and read across methods to predict the toxicity of untested contaminants can be used as the supplement to the hazard assessment of these projects. Though these methods are currently used more in research than in regulatory fields, the CFSA risk assessment team is cooperating with experts in computational toxicology from academic institutions to apply these methods into the risk assessment projects, e.g., efforts are being made in developing the methods involving the weight of evidence approach to evaluate the input values.

### 2.4 Threshold of toxicological concern (TTC)

TTC is the approach that can qualitatively assess the risk of a substance with little or no toxicological data. Having been developed by scientists for decades, TTC has been widely used in food safety risk assessment in China and other countries. EFSA published the guidance on the use of TTC in food safety assessment (Committee et al., 2019). Similarly, CFSA established the application guide of TTC in food safety risk assessment (CFSA, 2022). CFSA also developed the approach to apply TTC to the risk assessment of food contact materials and assessed the health risk of several PAHs, including bis (2-ethylhexyl) phthalate (DEHP), dimethyl 1,2-benzenedicarboxylate (DMP), and 1,2-benzenedicarboxylic acid diethyl ester (DEP) (Sui et al., 2012; Yong et al., 2016). The method to apply TTC for the safety assessment of non-intentionally added substances from food contact material was also developed (Zhong et al., 2017). In the last decade, CFSA has used TTC approach to assess the risk of Alternaria toxins in wheat, tomato, and citrus-based products, furfural and its derivatives in coffee products, and non-volatile compounds in polyamide food contact materials (Zhao et al., 2015a; Zhao et al., 2015b; Hu et al., 2021; Ji et al., 2022a; Liu et al., 2023a). Other academic institutions in China also adopted the TTC approach to assess the risk of flavor compounds in flavored milk (Chen et al., 2023), estrogens in milk products (Chen et al., 2014), fluorinated liquid-crystal monomers in foods (Yang et al., 2023), and mycotoxins such as Alternaria and Fusarium in different food categories (Fan et al., 2021; Zhou et al., 2022a; Ji et al., 2022b; Ji et al., 2022c; Qiao et al., 2022; Ji et al., 2023a; Ji et al., 2023b). Though TTC is not a “new” method, it remains a useful approach without testing, but with some limitations such as the method may not be applicable for some substances summarized in the guide by EFSA (Committee et al., 2019). Besides, there is currently no internationally coordinated internal TTC threshold that can be used in chemical assessments for multiple route-specific exposures (oral, dermal, inhalation) or aggregate exposures, which is also the direction of future research (Arnot et al., 2022).

### 2.5 Artificial intelligence

With the development of artificial intelligence (AI), AI approaches such as deep learning, machine learning, and neural network have potential to be applied in food safety, which has been extensively reviewed, including the main categories of data collection, data storage and transferring, data analysis, data visualization, and data security (Zhou et al., 2022b; Liu et al., 2023b). For the data-driven NGRA, AI has the potential to be used in the whole workflow, such as being applied in hazard assessment and exposure assessment in collecting and managing data.

To explore the application of AI-related methods in evidence management to build the AOP networks, EFSA developed the “Roadmap for actions on AI for evidence management in risk assessment” and launched the project “Exploring the use of AI for extracting and integrating data obtained through NAMs” (Bersani et al., 2022; Cavalli et al., 2022; Cattaneo et al., 2023). EFSA published a scientific report earlier this year showing the result of using AI-related tools for evidence management, such as automatic text summarization, keyword identification, deduplication, screening, and semi-automated characterization or classification (Cagnoni et al., 2023).

CFSA is also exploring the application of AI-related methods in evidence management for the NGRA, starting with developing a website with the function of semi-automatic comprehensive toxicological data searching and screening for a specific chemical contaminant. Once the website passes testing and validation, more functions will be added, including data extraction, data evaluation assistance (reliability and relevance evaluations), and data integration. The website is expected to assist the evidence-based food safety risk assessment projects. CFSA is also developing the application guide of AI in alternative protein risk assessment, attempting to be the starting point of using AI in a specific area of NGRA and expecting to guide the development of AI techniques in NGRA. The ongoing research areas of AI in CFSA include but not limited to evidence management, data prediction, and usage in other *in vitro* and *in silico* NAMs.

Research on AI in food safety risk assessment has also been conducted by academic institutions in China. Most of the research articles focused on developing AI models to predict the safety levels of certain hazards of different food products. The research team of Dong used AI methods such as neural networks and deep learning to predict the safety risk levels divided by clustering machine learning technique of contaminants such as heavy metals, veterinary drugs (florfenicol, enrofloxacin, and sulfonamide) residues, and arbofuran pesticide residues in different food categories (Jiang et al., 2022a; Jiang et al., 2022b; Dong et al., 2023; Lu et al., 2023). Wang et al. (2022) used the voting-ensemble deep learning method and the sub-models to analyze the risks of heavy metals in grain products. Xu et al. (2022b) combined three machine learning models to analyze multiple hazards (heavy metal, mycotoxin, pollutants) in rice. Other AI models used include agglomerative hierarchical clustering-radial basis function neural network integrating an analytic hierarchy process approach and the entropy weight, analytic hierarchy process integrated extreme learning machine based approach, and analytic hierarchy process based on the entropy weight and the autoencoder-recurrent neural network integrated framework for early warning analyses (Geng et al., 2017; Geng et al., 2021; Zhong et al., 2023). Additionally, AI methods were also used to generate virtual sample data expanding from a small dataset, using a model of improved random forest prediction method integrated Monte Carlo algorithm (Geng et al., 2022).

### 2.6 Omics

Covering a series of emerging technologies (genomics, transcriptomics, proteomics, metagenomics, epigenomics, metabolomics, exposomics, etc.), omics has been applied to the food safety risk assessment, and some omics-based approaches such as whole genome sequencing in genomics have been used in regulatory risk assessment of EFSA (Authority et al., 2018; Iacono et al., 2022). CFSA is also adopting omics-based approaches such as whole genome sequencing and nontargeted metabolomics in the ongoing food safety risk assessment projects. The application of whole genome sequencing in the surveillance of foodborne disease in China and the general use of nontargeted methods such as metabolomics have been reviewed (Shao et al., 2019; Li et al., 2021b). Other research studies in genomics by CFSA included the integration of whole genome sequencing and machine learning to identify antimicrobial resistance of *E. coli* (Peng et al., 2022), the complete genome and plasmid sequences *Salmonella enterica* subsp. *enterica* isolates carrying mcr-1 (Hu et al., 2019), and the complete genomic analysis of a mcr-1-carrying *Salmonella* Typhimurium strain from ready-to-eat pork (Wang et al., 2018), etc. The development and usage of omics databases, improvement and incorporation of various omics technologies in risk assessment are the possible research areas (Iacono et al., 2022).

### 2.7 Exposome

As one important component in food safety risk assessment, exposure assessment is essential for risk characterization. Though current exposure methods are sufficient for the risk assessments in specific scenarios, exposome data is needed to address the inter-individual variability for NGRA, e.g., epidemiological information is needed to include to analyze the diseased and healthy populations separately, and different exposure routes (oral, dermal, inhalation) and additional exposure pathways (e.g., occupational, environmental) should be considered (Wu, 2012; Wambaugh et al., 2019; Cattaneo et al., 2023). NAMs such as AI approaches can also be integrated with exposome to improve the exposure assessment (Wambaugh et al., 2019). The research gaps of exposome include the need of better causal inference of exposome data and adverse health effects, the consideration of physiologic variation, and the need for improved measurement and analysis methods (Escher et al., 2022).

CFSA has completed a project entitled “Characterizing the Exposome of Food Contamination and the China Total Diet Study.” Both the external and internal exposures of the contaminants recognized were analyzed and their correlation was studied through the development of the bioavailability, bioaccessibility, and PBTK modeling. The exposure biomarkers and the points of departure of the contaminants were also determined. The results of this project provided the data support for the food safety risk assessments in China (Lyu et al., 2022).

### 2.8 Integrated approaches to testing and assessment (IATA)

IATA is the approach to integrate, evaluate, and weigh the evidence from various sources, and it is preferred to be developed and used for NGRA, especially when risk assessments are carried out based on information generated from traditional methods and NAMs. OECD summarized the available guides of IATA and published related case studies (OECD, 2020; OECD). EFSA developed the guidance of weight of evidence approach in scientific assessments and developed case studies to improve the implementation of IATA in risk assessments (Hardy et al., 2017; EPoPP et al., 2021). CFSA risk assessment team is also developing the guide of IATA for NGRA, which will be the document guiding the application approaches of NAMs in the food safety risk assessment in China.

### 2.9 Adverse outcome pathway (AOP)

AOP includes the sequenced causally linked events at various biological organization levels between the exposure of a chemical and an adverse health effect, and it is the basis of IATA (Cattaneo et al., 2023; Viviani et al., 2023; OECD. Adverse). OECD published a guide on the use of AOP to develop IATA (OECD. Guidance, 2022). Through the establishment of AOP networks, it is expected to improve the understanding of molecular mechanisms and enhance regulatory implementation (Knapen et al., 2018; Villeneuve et al., 2018; Wiklund et al., 2023). EFSA developed AOPs to identify substances with endocrine disruptor properties (EPoPP et al., 2023; Viviani et al., 2023). The established AOPs are summarized on the website of OECD (OECD). AOPwiki is another platform with current available AOPs (validated or not) summarized, where AOP can be searched by molecular initiating events, adverse outcomes, key events, key event relationships, and prototypical stressors (AOP-Wiki 2023).

CFSA toxicology team has been working on the development of AOP based hazard assessment models at different biological organization levels. However, the other AOP related research in China focused more on the pharmacy and environment rather than food safety. To give a few examples, Chai et al. (2021) generated the AOPs of male reproductivity toxicity of inorganic arsenic; the research group of Wei studied different impairment mechanisms induced by black carbon, a typical environment pollutant, which contributed to the AOP of black carbon (Li et al., 2022b; Jiang et al., 2022c; Cui et al., 2023); Peng et al. (2021) overviewed the AOP of drug-induced liver injury. The collaboration between CFSA and the research institutions on the transformation of the related research into the field of food safety is the next step task of CFSA.

## 3 Problems and countermeasures

### 3.1 The establishment of NAMs development strategies applicable for food safety risk assessment in China

Facing the trend that the NGRA is based on NAMs, it is urgent to determine the roadmap for the short-term and long-term development of NAMs in China. As the only national level institution for food safety risk assessment in China, CFSA has the responsibility and is working on the establishment of the strategies to develop NAMs applicable for the risk assessments in China. The CFSA risk assessment team has been working in groups to develop the roadmaps for different NAMs. Principles of the strategies such as targeted NAMs with priority, development by stages and by layers are being considered. Once the strategies are determined, the document will be the basis to guide the development of the NAMs related technologies in China. Collaborating with research institutions in China, CFSA will guide and promote China’s development of NAMs to serve the development of NGRA to better ensure food safety in China, and to contribute to the NAMs for the international community as well.

### 3.2 The disciplinary development of modern food toxicology

In the 21st century, food toxicology is experiencing unprecedented development opportunities due to the progress in the life sciences. However, food toxicology still faces some special challenges, such as: how to accurately identify unknown hazards in food, food additives, or food-related products; how to fully simulate the long-term, mixed, oral, low-dose exposure characteristics of the human body to exogenous chemicals; and how to scientifically balance the health benefits of food ingredients with the risks. Solving these key problems depend on the continuous innovation and development of food toxicology itself as a discipline in addition to the progress in life sciences. Additionally, toxicity testing is currently shifting from traditional toxicology to more comprehensive systems toxicology research. However, systems toxicology also faces challenges in theory, technology, and application implementation. Its development requires interdisciplinary talents, high-throughput and high-content measurement technologies, powerful computer simulation capabilities and data analysis levels. Interdisciplinary and cross-field cooperation is needed to promote its theoretical and technological innovation and promote its application in practice.

Therefore, modern food toxicology needs to continuously adopt new technologies and methods from related fields such as molecular and cellular biology, analytical chemistry, statistics, and computer information science on the basis of traditional toxicological research, and integrate across disciplines to provide more accurate and efficient scientific support for toxicity assessment and risk assessment. CFSA will continue to work based on the “Food Toxicology Program” for the transition from traditional food toxicology to modern toxicology. The research on the NAMs in toxicology will be part of the work for the next stage.

### 3.3 The transformation from research to application of NAMs in NGRA

Currently, NAMs are more commonly used in research than in regulatory risk assessment. Though research progress of some NAMs such as alternative *in vitro* methods and computational toxicology has been made, most NAMs are not confident enough for the replacement of the current risk assessment methods. Besides CFSA, the scientific research institutions in China are also working on the NAMs. But different from CFSA whose direct purpose is to apply NAMs in the risk assessment, these institutions do not share the common goals or may not have research interests in risk assessments. CFSA keeps improving the collaboration with research institutions and has established the “1 + 6” cooperative mechanism with 6 leading universities in China. Taking advantage of the universities, CFSA will better lead the development of NAMs for NGRA with more communication and multiple collaborating mechanisms. On one hand, CFSA risk assessment team is organizing and implementing NAMs developing programs directly applicable for food safety risk assessment in urgent need. On the other hand, the related research of other collaborating institutions will be adopted and guided in risk assessment. Furthermore, application of the NAMs under research in food safety risk assessment projects is being conducted, which will facilitate the transformation of the emerging technology to the risk assessment in phases. The problems met during the application attempts will guide the changes of the research focus of the NAMs in return.

To meet the needs of toxicity testing technology development, the CFSA research team of the Toxicology Program has also established targeted NAMs research directions, including alternative methods, toxic pathways, and extrapolation models. To continue the research and promote the transformation of these methods into NGRA, the next steps are: 1) continue to accelerate the research and development of hazard identification technologies based primarily on human-derived cells and zebrafish *in vitro*, while exploring biological carriers that are closer to human target organs based on previous alternative model research; 2) make full use of modern toxicological technologies such as genomics, transcriptomics, proteomics, and metabolomics as well as data integration and analysis for the discovery of toxic pathways and the explanation of toxic mechanisms, and develop high-content and high-throughput hazard identification technologies based on key molecular characteristics of toxicity; 3) combine structure-activity analysis, PBTK-QIVIVE, and other methods to further improve the hazard assessment system. At the same time, gradually promote the construction of efficient platforms such as laboratory intelligence and digitization to effectively support the implementation of national strategies for food safety.

### 3.4 The continuous construction of the talent team in modern food toxicology

Starting from the 1950s, the modern food toxicology of China formed a relatively systematic toxicology discipline, with a large number of experts and technical backbones in the field of food toxicology. However, in recent years, the research, update, and application of novel technologies and methods required more trained toxicologists, and the technical strength and talent team building are worrying. It is urgent to strengthen the construction of professional teams in food toxicology in China to provide better human resources for food safety risk assessment. The development of NAMs relies on an adequate number of professional toxicologists. The increase in capacity and the improved technical training are the keys in the talent team construction.

## 4 Summary

In summary, CFSA is leading the development of NAMs for NGRA. The “Food Toxicology Program” has always focused on the core work of CFSA, practiced the concept of translating toxicological research, carried out key technical support work, and provided a solid toxicological database and scientific basis for food safety risk assessment and relevant standard formulation and revision. Not only promoting the traditional food toxicology, the “Food Toxicology Program” is also the key program for the development of NAMs of toxicology.

Based on the related research and the practical needs, CFSA is working on the strategies to develop NAMs for NGRA in China. Collaborating with other research institutions, CFSA will guide the development of NAMs in China and integrate the efforts of different institutions to develop the NAMs applicable for the regulatory food safety risk assessment.
